# Malaria, Moderate to Severe Anaemia, and Malarial Anaemia in Children at Presentation to Hospital in the Mount Cameroon Area: A Cross-Sectional Study

**DOI:** 10.1155/2016/5725634

**Published:** 2016-11-08

**Authors:** Irene Ule Ngole Sumbele, Sharon Odmia Sama, Helen Kuokuo Kimbi, Germain Sotoing Taiwe

**Affiliations:** ^1^Department of Zoology and Animal Physiology, University of Buea, Buea, Cameroon; ^2^Department of Medical Laboratory Sciences, University of Bamenda, Bamenda, Cameroon

## Abstract

*Background*. Malaria remains a major killer of children in Sub-Saharan Africa, while anaemia is a public health problem with significant morbidity and mortality. Examining the factors associated with moderate to severe anaemia (M*d*SA) and malarial anaemia as well as the haematological characteristics is essential.* Methodology*. Children (1–14 years) at presentation at the Regional Hospital Annex-Buea were examined clinically and blood samples were collected for malaria parasite detection and full blood count evaluation.* Results*.* Plasmodium falciparum*, anaemia, and malarial anaemia occurred in 33.8%, 62.0%, and 23.6% of the 216 children, respectively. Anaemia prevalence was significantly higher in malaria parasite positive children and those with fever than their respective counterparts. M*d*SA and moderate to severe malarial anaemia (M*d*SMA) were detected in 38.0% and 15.3% of the participants, respectively. The prevalence of M*d*SA was significantly higher in children whose household head had no formal education, resided in the lowland, or was febrile, while M*d*SMA was significantly higher in febrile children only. Children with M*d*SMA had significantly lower mean white blood cell, lymphocyte, and platelet counts while the mean granulocyte count was significantly higher.* Conclusion*. Being febrile was the only predictor of both M*d*SA and M*d*SMA. More haematological insult occurred in children with M*d*SMA compared to MdSA.

## 1. Background

In spite of the increase in control measures and reported 18% and 48% decline in the number of malaria cases and deaths, respectively, globally between 2000 and 2015, malaria remains a major killer of children especially in Sub-Saharan Africa, taking the life of a child every 2 minutes [1]. However, in order to properly evaluate control measures, regular updates of disease morbidities in public health services and community settings in the country are invaluable. Even though studies have been carried out on severe and uncomplicated malaria in children admitted to hospitals in different parts of the country [2, 3] as well as uncomplicated and asymptomatic malaria in the communities [4] and primary school children [5], there is a dearth of information on malaria-related morbidities at presentation in the general medical outpatient department in the country.

While malaria is one of the factors that contributes to the public health problem of anaemia in children in Cameroon [2, 6, 7], in almost all countries in Sub-Saharan Africa, anaemia is a moderate or severe public health problem causing significant morbidity and mortality [8]. Much of the burden of infections operates through the mechanism of anaemia which is characterized by a reduction in haemoglobin concentration causing impairment in meeting the oxygen demands of the body. In African children this haematological state is determined by combinations of nutritional deficiencies, infectious diseases (malaria, hookworm infections, and human immunodeficiency virus infections), and the genetic constitution of red cell haemoglobin [9–12]. However, WHO malaria report [1] stated that, in most malaria endemic areas, less than half of patients with suspected malaria infection are truly infected with a malaria parasite. Consequently, parasitological confirmation by light microscopy or rapid diagnostic tests before the commencement of treatment, in children in the outpatient department, is invaluable. Additionally, this provides an opportunity to evaluate the burden of malaria and the prevalence of anaemia and its severity in febrile children. The findings might serve as a predictor of malaria-related mortality.

Malarial anaemia (MA) is a multifactorial disease for which the complex etiological basis is only partially defined. Severe MA is one of the main clinical presentations of severe malaria caused by* P. falciparum* [2]. The aetiology of severe MA in malaria endemic areas may include a number of discrete as well as overlapping features, such as lysis of infected and uninfected RBCs [13], splenic sequestration of RBCs [14], dyserythropoiesis and bone marrow suppression [15], infectious diseases, and chronic transmission of malaria. While haematological insults resulting in moderate and severe anaemia in infection with* Plasmodium falciparum* have been established [16], the exact differences in the pathophysiology of anaemia in the various clinical settings, ages, and geographic areas are poorly defined [17].

In children presenting at a hospital in western Kenya, wasting was associated with increased presentation of MA. In addition the caretakers level of education and occupation significantly correlated with anaemia and MA [12]. On the other hand, hospital based studies in the Mount Cameroon area indicated severe MA as the main clinical presentation of severe malaria but did not examine sociodemographic or nutritional factors associated with the presentation [2]. Hence, assessing the influence of some sociodemographic and nutritional indices on the prevalence of M*d*SA and MA in children will provide valuable information to the health authorities. This will enable informed decision and the appropriate allocation of scarce resources for proper child health management and control of these morbidities. This study was undertaken to explore the hypothesis that sociodemographic factors and nutritional indices influence the presentation of children in the outpatient department with M*d*SA and M*d*SMA. The objectives of the study therefore were to determine the prevalence of* falciparum* malaria, MA, M*d*SA, and M*d*SMA in children at presentation for consultation in general medical outpatient department, evaluate the attributable risk of anaemia caused by malaria, and assess the variation in haematological indices in moderate to severe anaemic and malarial anaemic children.

## 2. Materials and Methods

### 2.1. Study Area

This study was carried out in the Regional Hospital Annex-Buea, Fako Division, South West Region. Buea, a town in the Mount Cameroon area, is situated at latitude 3°57′–4°27′N and longitude 8°58′–9°25′E, 500–4080 metres above sea level (asl) and is located on the southeast slope of Mount Cameroon. Buea has an estimated population of above 200.000 inhabitants constituting essentially of the Bakweri indigenes in the villages and a highly cosmopolitan population in the urban space with the indigenes at a minority [18]. The climate in Buea tends to be humid, with temperatures varying from 18°C to 27°C, average relative humidity of 80%, and average rainfall of 4000 mm. There are two seasons, the rainy and the dry seasons, which start from mid-March to October and November to mid-March, respectively. The prevalence of malaria parasitaemia in the Mount Cameroon area varies from 60.6%, in lowland altitude, to 7.7% in the highlands [5].

### 2.2. Study Population

The study population included children of both sexes aged 1–14 years, who presented themselves at the Regional Hospital Annex-Buea for consultation during the period of study. Children who participated in the study came from various localities and altitudes. The altitude was classified as lowland (0–167 m asl), middle belt (600–650 m asl), and highland (897–918 m asl) as reported by Kimbi et al. [5]. Only children whose parent/guardian signed the informed consent/assent forms following the education on the importance of the study were enrolled. For the purpose of comparability, patients with a history of antimalarial treatment in the preceding two weeks or who had a blood transfusion three months prior to the start of study or had haemoglobin genotype SS were not enrolled in the study.

### 2.3. Study Design

This cross-sectional hospital based study was carried out during the peak malaria transmission season from the month of May to August 2014, in the Regional Hospital Annex-Buea. Following administrative clearances and ethical approval for the study, informed consent/assent forms explaining the purpose, risks, and benefits of the study were given to parent or guardian. After obtaining consent/assent from the participant, clinical evaluation was carried out and a questionnaire was administered prior to blood sample collection. Blood sample was collected from each child for determination of malaria parasite status and full blood count evaluation. The optimum sample size was calculated using the prevalence of* P. falciparum *parasitaemia in the region of 36.6% [4] and anaemia prevalence in a hospital based studies in the Mount Cameroon area of 94.7% [2]. The sample size was determined using the formula *n* = *z* ^2^ *pq*/*d* ^2^ [19], where *n* represented the sample size required; *z* was 1.96, which is the standard normal deviate (for a 95% confidence interval, CI); *p* values were 36.6% and 94.7%, the proportion of malaria parasitaemia and anaemia prevalence, respectively; *q* was 1 − *p*; and *d* was 0.05, the acceptable error willing to be committed. The optimum sample size obtained from the average of both sample sizes was 217.

### 2.4. Ethical Consideration

Ethical clearance document for the study was obtained from the Institutional Review Board hosted by the Faculty of Health Sciences, University of Buea (2014-04-0261/UB/FHS/IRB) following administrative clearance from the Regional Delegation of Public Health. Informed consent/assent forms were given or read and explained to parents or guardians of the study participants at presentation. The consent/assent forms stated the purpose and benefits of the study and the amount of blood to be collected from each child. Emphasis was laid on the voluntary participation of the children in the study and on the point that their refusal to participate in the study will in no way affect the treatment quality the children were to receive. Only those who signed the consent/assent forms were enrolled into the study. The parents or guardians were free at any point in time to stop the participation of the child/children in the study.

### 2.5. Clinical Evaluation

For each child a general clinical evaluation was carried out by the medical personnel in charge. The axillary temperature was measured using a digital thermometer and fever was classified as temperature ≥ 37.5°C. Symptoms and the duration of the symptoms were recorded. Anthropometric parameters such as height and weight were measured using a measuring tape and a weighing scale, respectively. Height-for-age (HA), weight-for-age (WA), and weight-for-height (WH) standard deviation (SD) scores (*z* scores) were computed based on the National Centre for Health Statistics- (NCHS-) WHO growth reference curves using the nutrition module of the Epi Info 2000 program [20]. Underweight was defined as weight-for-age *z* (WAZ) score of <−2; wasting was defined as a weight-for-height *z* (WHZ) score of <−2; and stunting was defined as height-for-age *z* (HAZ) score of <−2. A child was identified as being malnourished if he or she scored <−2 in one of the anthropometric indices of HA, WA, and WH [21]. The spleen was felt at the tip by pressing the abdomen under the left costal border and splenomegaly was graded according to Hackett's classification [22].

### 2.6. Questionnaire

A structured questionnaire was administered to parent or guardian of the child in order to obtain information on demography, socioeconomic status (SES), type of accommodation, health-seeking behaviour, access to health facilities, malaria control measures, knowledge on the signs of anaemia, and feeding practices. The socioeconomic status was classified as poor, average, and rich as described by Kimbi et al. [5] and Sumbele et al. [23].

### 2.7. Laboratory Methods

Venous blood samples (about 4 mL) were collected using sterile disposable syringes from children whose parent or guardian signed the assent forms. Thin and thick blood films were prepared and the remaining blood sample dispensed into labelled ethylenediaminetetraacetate (EDTA) tubes. Labelled blood samples were transported on ice in a cool box of temperature between 8 and 10°C to the University of Buea Malaria Research Laboratory for further analyses. The thick and thin blood films prepared on glass slides at the time of blood sampling were stained with Giemsa and examined following standard protocols [24]. Parasite density was determined based on the number of parasites per 200 leukocytes on thick blood film with reference to participants' white blood cell count. If gametocytes were seen, the count was extended to 500 leukocytes [25]. Parasitaemia was categorised as low (<1,000 parasites/*μ*L blood), moderate (1,000–4,999 parasites/*μ*L blood), high (5,000–99,999 parasites/*μ*L blood), and hyperparasitaemia (≥100,000) [4, 5, 26]. A complete blood count including values for white blood cell (WBC), red blood cell (RBC) and platelet counts, haemoglobin concentration (Hb), haematocrit (Hct), mean corpuscular volume (MCV), mean corpuscular haemoglobin (MCH), mean corpuscular haemoglobin concentration (MCHC), mean platelet volume (MPV), red cell distribution width (RDW), platelet distribution width (PDW), red blood cell distribution width coefficient of variation (RDW-CV), and red blood cell distribution width standard deviation (RDW-SD) was obtained using an autohaematology analyser, the Beckman Coulter counter (URIT 3000), following the manufacturer's instructions. Anaemia was defined as Hb < 11.0 g/dL [27] and further classified as described by Cheesbrough [24] as severe (Hb < 7.0 g/dL), moderate (Hb between 7.0 and 10.0 g/dL), and mild (>10 g/dL Hb <11 g/dL). Malarial anaemia (MA) was defined as children with a malaria-positive smear for* P. falciparum* parasitaemia (of any density) and Hb < 11 g/dL. Moderate to severe anaemia was defined as Hb < 10 g/dL and moderate to severe malarial anaemia defined as malaria parasite positive + Hb < 10 g/dL.

### 2.8. Statistical Analysis

Data collected was cleaned up and analysed using the IBM-Statistical Package for Social Sciences (IBM-SPSS) version 20. Continuous variables were summarized into means and standard deviations and categorical variables reported as frequencies and percentages were used to evaluate the descriptive statistics. The differences in proportions were evaluated using Pearson's Chi-Square (*χ* ^2^) and the bivariate associations between haematological values and malaria parasite density by Pearson's rank correlations (*r*). Group means were compared using analysis of variance (ANOVA), Student's *t*-test, or Kruskal Wallis test where appropriate. Parasite density was log transformed before analysis. A multinomial logistic regression model analysis was conducted to evaluate potential determinants of M*d*SA and M*d*SMA with age, sex, SES, level of education, altitude, fever, and nutritional status as independent variables. The odd ratios (OR) computed was used to evaluate the risk factors. The attributable risk (AR) of anaemia caused by malaria (AR%) was calculated accordingly [28]: [(*n*_1_ *m*_0_–*n*_0_ *m*_1_)/*n*(*n*_0_ + *m*_0_)] × 100, where *n*_0_ = anaemic children without malaria and *n*_1_ = anaemic children with malaria, whereby *n*_0_ + *n*_1_ = *n*, *m*_0_ = nonanaemic children without malaria, and *m*_1_ = nonanaemic children with malaria, whereby *m*_0_ + *m*_1_ = *m*. Significant levels were measured at 95% confidence interval (CI) with significant differences set at *P* < 0.05.

## 3. Results

### 3.1. Characteristics of Participants

The consent of 254 children at presentation to the general outpatient department in the Regional Hospital Annex-Buea was sought for their participation in the study of the burden of malaria, M*d*SA, and M*d*SMA. Complete clinical and laboratory data for a total of 216 (85%) children, with a mean age of 6.3 ± 4.1 (range = 1–14) years, were included in the cross-sectional study. Majority of the parents/guardians of the children had some knowledge about malaria (94.4%) and anaemia (75.8%) and more than half of the children ate fruits (58.3%) and vegetables (56.5%) regularly.

As revealed in Table 1, 52.3% (113) of the participants were males and 47.7% (103) were females. Majority of the children resided in the middle belt (75.6%) of the Buea municipality, were from homes of average socioeconomic status (50.0%), and their parents/caregivers had at least secondary level education (43.5%). The proportion of mosquito bed net (MBN) use in the studied population was 53.2% with comparable usage amongst the different age groups and sexes.

Fever, splenomegaly, malarial anaemia, and malnutrition were observed in 49.5%, 22.2%, 23.6%, and 24.1% of the children, with no significant differences in age and sex. The prevalence of anaemia in the studied population was 62.0%, with children of the 1–5 years age group having the highest occurrence (67.6%) compared to their counterparts. The difference was statistically significant (*P* = 0.036). Wasting occurred in 6.0% of the children. While the prevalence of wasting was significantly higher (*P* = 0.02) in females (10.7%) than males (1.8%), a comparison of the different age groups revealed no significant difference (Table 1).

### 3.2. *Falciparum* Malaria

**Figure 1:**
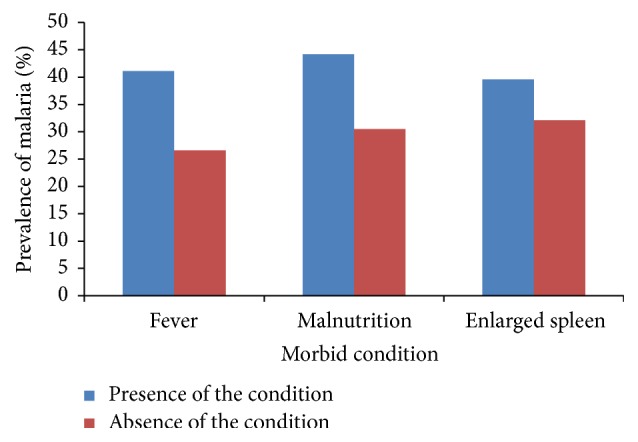
Prevalence of malaria parasite as affected by the status of fever, malnutrition, and splenomegaly. Presence of a morbid condition refers to (i) presence of fever (children with temperature ≥ 37.5°C) and (ii) presence of malnutrition (children with <−2 *z* scores in one of the anthropometric indices (HA, WA, and WH)) and (iii) presence of enlarged spleen (children with enlarged spleen). Absence of a condition refers to (i) absence of fever (children with body temperature < 37.5°C), (ii) absence of malnutrition (well nourished children with >−2 *z* score in all anthropometric indices), and (iii) absence of enlarged spleen (children with normal spleen size).

*Plasmodium falciparum* occurred in 33.8% (73) of the 216 children at presentation with no significant difference in sex and age. Although not significant, children of the 1–5 years age group and males had the highest geometric mean parasite density (GMPD)/*μ*L of blood compared to their counterparts as shown in Table 1. The prevalence of malaria was highest in patients from the lowland (47.4%, 9) compared to their counterparts from the middle belt (32.3%, 32) and highland (33.3%, 12) although the difference was not statistically significant (*χ* ^2^ = 1.73, *P* = 0.42). A greater proportion of the children had high parasite densities (45.2%, 33) while low, moderate, and hyperparasitaemia occurred in 28.8% (21), 17.8% (13), and 8.2% (6) of them, respectively. In addition, the prevalence of malaria was significantly higher (*χ* ^2^ = 5.09, *P* = 0.024) in children with fever (41.1%) when compared with those with no fever (26.6%) at presentation. On the other hand, the high prevalence of malaria observed in malnourished children (44.2%) and those with enlarged spleens (39.6%) compared to their counterparts was not statistically significant as shown in Figure 1.

### 3.3. Monthly Prevalence

**Figure 2:**
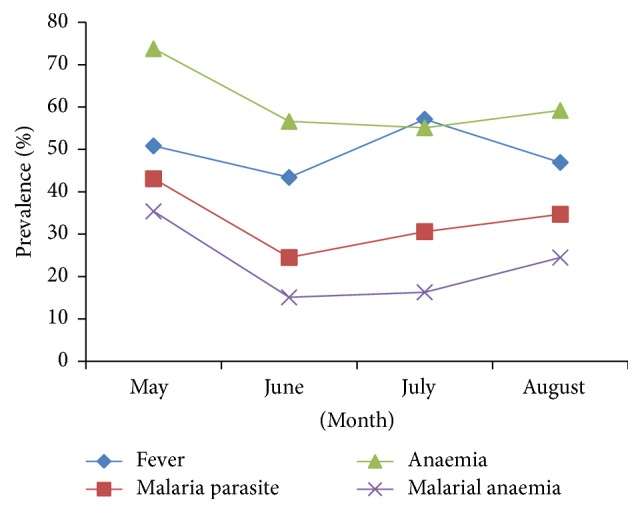
Monthly prevalence of fever, malaria parasite, anaemia, and malarial anaemia during the study period.

The prevalence of fever was highest in the month of July (57.1%), while malaria parasite, anaemia, and MA were highest in the month of May (43.1, 73.8, and 35.4%, resp.) as shown in Figure 2. However, only the monthly difference in prevalence of MA was statistically significant (*χ* ^2^ = 8.59, *P* = 0.035).

### 3.4. Anaemia and* Falciparum* Malaria/Fever

**Figure 3:**
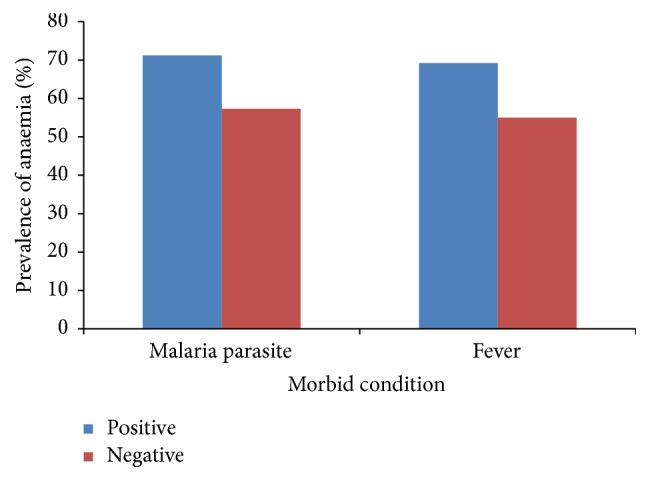
Prevalence of anaemia as affected by malaria parasite and fever status. Positive denotes (i) positive for malaria parasite (participants with positive slide for malaria parasite) and (ii) positive for fever (participants with temperature ≥ 37.5°C). Negative denotes (i) negative for malaria parasite (participants with negative slide for malaria parasite) and (ii) negative for fever (participants with temperature < 37.5°C).

**Figure 4:**
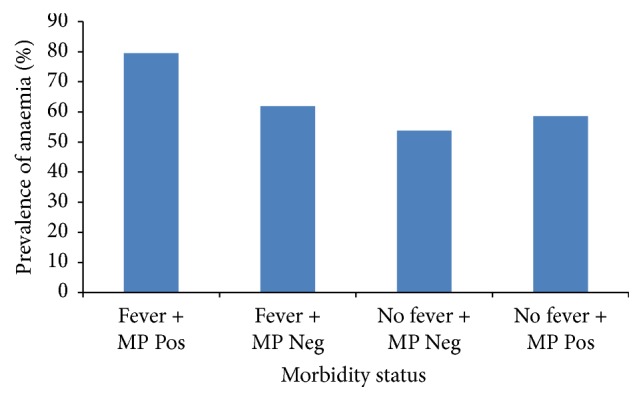
Prevalence of anaemia as influenced by morbidity status. MP Pos = malaria parasite positive. MP Neg = malaria parasite negative. Comparison of participants with fever + MP positive and those with fever and MP negative (*χ* ^2^ = 3.78, *P* = 0.052). Comparison of participants with no fever + MP negative versus those with no fever + MP positive (*χ* ^2^ = 0.20, *P* = 0.651).

As shown in Figure 3, children who were malaria parasite positive had significantly higher (*χ* ^2^ = 3.96, *P* = 0.047) prevalence of anaemia than those negative. Similarly, the prevalence of anaemia was significantly higher (*χ* ^2^ = 4.57, *P* = 0.033) in children who presented with fever than their nonfeverish counterparts. The prevalence of anaemia as influenced by the morbid state is shown in Figure 4. Children with both fever and detectable malaria parasitaemia had a higher prevalence of anaemia (79.5%) that approached significance when compared with those with fever only (61.9%).

### 3.5. Moderate to Severe Anaemia and Malarial Anaemia

Mild, moderate, and severe anaemia were prevalent in 23.6%, 29.6%, and 8.3% of the children, respectively, with no significant difference in sex and age. At presentation for consultation, M*d*SA and M*d*SMA were detected in 38.0% and 15.3% of the children, respectively. While the prevalence of M*d*SA and M*d*SMA was comparable amongst age group, gender, SES, family size, splenic, and nutritional status, and statistically significant differences were observed with the level of education of head of household (*P* = 0.046), altitude of residence (*P* = 0.012), and fever status (*P* = 0.019 and *P* = 0.012) as shown in Table 2. Explicitly, in comparison with their contemporaries, the prevalence of M*d*SA was significantly higher in children who came from homes where the head of household had no formal education (60.3%), resided in the lowland (63.2%), or had fever (45.8%). On the other hand, M*d*SMA was significantly higher in children who had fever only (21.5%) when compared with their corresponding equivalents.

The multinomial logistic regression model demonstrated that the altitude, more specifically the lowland (*P* = 0.02), and being febrile (*P* = 0.016) were significant predictors of M*d*SA, while being febrile (*P* = 0.016) was the only significant factor associated with M*d*SMA as shown in Table 3.

### 3.6. Attributable Risk of Anaemia due to Malaria

The AR of anaemia caused by malaria in the studied population was 7.6% and this was higher in females (12.0%) than males (4.4%) and in children of the 6–10 years age group (10.1%) than the 1–5 years (3.2%) and the 11–14 years age group (5.6%). On the other hand, the AR of moderate to severe anaemia caused by malaria was 9.4% with that of females (16.3%) being higher than males (2.2%). In addition, the AR of moderate to severe anaemia due to malaria was higher in children of the 11–14 years age group (36.0%) compared to the 1–5 years (7.2%) and the 6–10 years (7.2%) ones.

### 3.7. Haematological Indices

Correlations between haematological values and malaria parasite density revealed a significant negative association between malaria parasite density and lymphocyte count percentage (*r* = −0.239, *P* = 0.041) while a significant positive relationship was observed between granulocyte count % and malaria parasitaemia (*r* = 0.254, *P* = 0.03). A nonsignificant negative trend was observed between platelet count and malaria parasitaemia (*r* = −0.133, *P* = 0.267). Findings from the study revealed significantly lower WBC counts (*P* = 0.037), lymphocyte % (*P* = 0.004), and platelet counts (*P* = 0.04) in children with M*d*SMA when compared with those with M*d*SA. On the other hand, children with M*d*SMA had significantly higher (*P* = 0.033) granulocyte % than those with M*d*SA (Table 4). Although not statistically significant the mean RBC, Hb, Hct, PDW, and RDW-SD were lower in those with M*d*SMA than those with M*d*SA.

## 4. Discussion

This cross-sectional study examines* P. falciparum* malaria, moderate to severe anaemia, and malarial anaemia as public health problems in children ≤14 years at presentation in the general medical outpatient department in the Regional Hospital Annex-Buea, Mount Cameroon area. The overall malaria parasite prevalence of 33.8% observed by microscopy is higher than the overall 29.8% reported for children reporting to the Mbakong Health Centre, in the North West Region of Cameroon, between 2006 and 2012 [3] and the 29% observed in febrile outpatients that visit either a public or a private health facility or medicine retail store in Bamenda and Yaoundé [29]. The equatorial climate in the Mount Cameroon area is characterized by abundant rainfall and constant humidity, all of which are factors that favour intense and perennial transmission of the malaria parasite [2], which may be a contributing factor in the higher prevalence of malaria in spite of the intensification of control measures.

Against the backdrop of a decline, the increased prevalence of malaria parasite observed with age culminating in children of the 11–14 years age group having the highest prevalence (43.2%) and a greater presence of high parasite densities (45.2%) signals a change in burden. Similar observation of a change in malaria morbidity following control measures had been reported earlier in the region [4]. While this trend may be linked to the effective use of mosquito bed nets observed in children of the 1–5 years age group when compared with the other age groups, the significantly higher prevalence of malaria in febrile children highlights fever as one of the symptoms characteristic of malaria infection. Similar significant association between malaria and fever has been reported in the same region [5, 30] even though fever may be a poor indicator of malaria where infection with other pathogens is possible [31]. Nevertheless the confirmed malaria case in febrile children (38.2%) is similar to the trend observed in Ethiopia [32].

Findings from the study revealed an anaemia prevalence of 62% with the highest prevalence in children of the 1–5 years age group. This observation is comparable to the national value of 60% reported in 2011 [33] but lower when compared with the >73.5% observed in febrile Gabonese children in a health facility based survey [34]. The significantly higher prevalence of anaemia in feverish children compared to nonfeverish ones is in line with other studies [4, 7, 34, 35]. Similarly, the significantly higher prevalence of anaemia in malaria-positive individuals is consistent with findings of several studies [34, 36, 37]. However, worthy of note, the 71.2% prevalence of anaemia observed in children with malaria parasitaemia and 79.5% in children who were both febrile and malaria parasite positive is lower than 94.7% and 80.3% obtained by Achidi et al. [2] and Sumbele et al. [7], respectively, in the same region. This regional drop in prevalence of anaemia in children with malaria may be accredited to the sustained malaria control measures and its impact on the outcome measure. More so, frequent research and sensitization campaigns carried out in this study area raise awareness on the condition.

Observation from the study revealed no significant differences in the categorisation of anaemia (severe, mild, and moderate) when the various groups were compared. Hence, the moderate and severe groups of anaemia were lumped together for comparison with such other studies. However, the intention of the paper was to report on moderate to severe anaemia with or without malaria. Findings revealed a 38.0% prevalence of moderate to severe anaemia not associated with malaria and its occurrence was found to be significantly associated with fever. Furthermore, the logistic regression analysis revealed febrile children were two times at odds of being moderate to severely anaemic than their afebrile counterparts. This is not unusual as fever is not specific to infection with malaria parasite only but could be indicative of other anaemia-causing infections [38]. As with anaemia, the significantly (*P* = 0.046) higher prevalence of M*d*SA in children whose parent/guardian/head of household had no formal education corroborates the findings of Oliveira et al. [39]. Lower levels of education may lead to lower paid work, hence less access to quality food, goods, and services that are beneficial to a child's health [40]. On the other hand, above secondary schooling may facilitate better feeding practices and habits gained through knowledge on nutritional composition of foods and health education.

The highest prevalence of M*d*SA observed in children who came from the lowland (63.2%) in contrast to the lowest prevalence observed in high altitude dwellers (22.2%) is not surprising given that haemoglobin concentration increases with altitude [27] and the lowland has favourable environmental and climatic conditions which may promote the rapid growth of the anopheline vectors and consequently a high rate of malaria transmission [5, 41] in the area. This cohort of children may have been exposed to the impact of repeated infections with malaria parasites even though they were negative at the time of examination. However, in a combination of factors in the logistic regression analysis, our findings revealed to a certain extent a significant protection against moderate to severe anaemia in children from the lowland.

The prevalence of M*d*SMA (15.3%) is comparable to the 13% recorded in Mozambican children [42]. In the context of a decline in the prevalence of malaria in the region, the moderate prevalence of malarial anaemia (23.6%) and the low occurrence of M*d*SMA at presentation probably suggest that malaria is not the major contributing factor to the public health problem of anaemia. Even though the study had as limitation the length of the period in which the investigation was carried out and the number of children examined, that notwithstanding, the investigation was carried out during the peak season of malaria transmission to ensure probability of encountering malaria parasite positive cases. However, the contribution of malaria to the public health problem of anaemia should be interpreted with caution and further study in different ecological settings and a larger population should be carried out.

In conformity with Leite et al. [43] who stated that children in household with four to five children and nine or more total residents were prone to anaemia, children who lived in homes with >10 persons had a higher prevalence of M*d*SA and M*d*SMA than their counterparts. The constraints of large family size may not only be the number of individuals to provide adequate diet in terms of nutrients and proportions, but the lack of resources to provide adequate health care such as providing appropriate malaria treatment. Delayed treatment-seeking and inappropriate medication, both of which are common among people in the Mount Cameroon area, have been reported earlier as risk factors of anaemia [7].

The low estimate of the risk of anaemia (7.6%) and moderate to severe anaemia (9.4%) that may be attributed to malaria in the children at presentation clearly indicate the important contribution of other inflammatory infections or diseases. Why females had a higher attributable risk of anaemia and moderate to severe anaemia due to malaria is unclear as no significant gender differences were observed in prevalence and density. Even though studies on the attributable risk of anaemia and moderate to severe anaemia due to* falciparum* malaria have seldom been carried out in Cameroon, previous studies in the Mount Cameroon area, in apparently healthy children, revealed a moderate (24.5%) contribution of* falciparum* malaria to anaemia [23]. Inferences from both studies most likely indicate the notion that of greater importance is the contribution of asymptomatic* P. falciparum* infection to the public health problem of anaemia compared to falciparum infection in febrile children. However, out of the ordinary is the major contribution of* falciparum* malaria to moderate to severe anaemia (36.0%) in children of the 11–14 years group which cannot be ignored.

The significant negative correlation observed between parasite density and lymphocyte percentage as well as the significantly lower lymphocyte counts in children with M*d*SMA is not unusual. Previous studies have reported a decrease in lymphocyte counts in malaria infection [36, 44]. On the other hand, in line with Lucien et al. [45], findings from this study revealed a significantly higher mean granulocyte counts in children with M*d*SMA with a significant positive relationship between parasite density and the percentage of granulocytes (*P* = 0.02). Although lymphocytosis has been reported elsewhere [46] the negative relationship observed may be attributed to the role lymphocytes, particularly T cells, play in malaria immunity and probably reflect redistribution of lymphocytes with sequestration in the spleen [47, 48].

The nonsignificant lower values of RBC, Hb, and Hct observed in children with M*d*SMA compared to M*d*SA reflect the haematological abnormalities which is a hallmark of* falciparum* malaria earlier reported by several studies [16, 36]. However, the lack of any significant differences in red cell indices probably indicates that the erythropoietic response to infection between those with M*d*SMA and M*d*SA may not have been different. This may be apparent in the similarities in values of markers (RDW-SD and RDW-CV) of ineffective erythropoiesis of bone marrow [49] and inflammation [50] which were within the normal range.

The association between platelet counts and malaria parasite has been reported by several studies [5, 16]. The lower than normal mean platelet counts observed in children with M*d*SMA (143.7 × 10^9^/*μ*L as against 233.5 × 10^9^/*μ*L) clearly highlight the common occurrence of thrombocytopenia (57.6%) in this cohort of children. The high presence of thrombocytopenia may be considered as a marker of disease severity as indicated by Maina et al. [16] and not parasite burden as no association was observed between platelet counts and parasite density. Albeit studies in Nigeria [51] revealed children with low platelet counts were likely to have anaemia, findings from this study reveal no significant association between platelet counts and Hb or parasite density. While observations from the study showed similarities in MPV in children with M*d*SMA and M*d*SA, the reason why the negative correlations between MPV and platelet counts in children with M*d*SMA was not significant (*r* = −0.116, *P* = 0.528) while that of M*d*SA was significant (*r* = −0.240, *P* = 0.031) requires further investigation to explain the difference.

Although hospital based survey is a limitation of the representation in the general population, these findings represents an approximation of the morbidity and factors related to moderate to severe anaemia and malarial anaemia which can be applicable in a wider population with different settings although with caution. There was a limitation in the diagnostic facilities of other causes of anaemia such as bacterial or viral infections and inherited haemoglobinopathies such as thalassemias, which are known to cause or are contributing factors in the aetiology of anaemia. Hence their confounding influence could not be ascertained.

## 5. Conclusions

Even with the decline,* falciparum* malaria is a public health problem with higher occurrence in children presenting with fever and those of the 11–14 years age group. The prevalence of anaemia is high in febrile and malaria parasite positive children with moderate to severe anaemia being a moderate public health problem. The factors significantly associated with M*d*SA include residing in the lowland and being febrile at presentation while being febrile was the only factor significantly associated with M*d*SMA. The AR of anaemia due to malaria was low; however, children of the 11–14 years age group had the highest risk of moderate to severe anaemia attributable to malaria. Haematological insult with a significant reduction in WBC, lymphocyte, and platelet counts occurred in children with M*d*SMA. To assert these findings the cross-sectional study needs to be extended in period and facilities to draw broad base conclusions.

## Figures and Tables

**Table 1:** Sociodemographic, altitude, and clinical characteristics of the participants by age and sex.

Parameters	Age groups in years			Gender		Total
	1–5	6–10	11–14	Male	Female	
% (*N*)	51.4 (111)	23.2 (61)	20.4 (44)	52.3 (113)	47.7 (103)	100 (216)
Mean age (SD) in years	2.8 (1.3)	7.8 (1.4)	12.7 (1.2)	6.1 (4.0)	6.5 (4.3)	6.3 (4.1)
Mean weight (SD) in Kg	14.9 (3.8)	26.0 6.1)	45.6 (11.8)	23.5 (14.8)	25.1 (14.8)	24.3 (13.6)
Mean height (SD) in cm	90.3 (15.1)	124.2 (14.0)	154 .7 (11.0)	111.5 (28.9)	114.7 (29.6)	113.0 (29.2)
SES						
Poor	31.5 (25)	34.4 (21)	31.8 (14)	30.1 (34)	35.0 (36)	32.4 (70)
Average	55.9 (62)	42.9 (30)	36.4 (16)	53.1 (60)	46.6 (48)	50 0 (108)
Rich	12.6 (14)	16.4 (10)	31.8 (14)	16.8 (19)	18.4 (19)	17.6 (38)
Education level of Parent/caregiver						
No formal (*n*)	2.7 (3)	3.3 (2)	0.0 (0)	2.7 (3)	1.9 (2)	2.3 (5)
Primary (*n*)	19.8 (22)	16.4 (10)	11.4 (5)	16.8 (19)	17.5 (18)	17.1 (37)
Secondary (*n*)	43.2 (48)	41.0 (25)	47.7 (21)	39.8 (45)	47.6 (49)	43.5 (94)
Tertiary (*n*)	34.2 (38)	39.3 (24)	40.9 (18	40.7 (46)	33.0 (34)	37.0 (80)
Altitude of residence						
Lowland (*n*)	7.2 (8)	9.8 (6)	11.4 (5)	7.8 (8)	10.7 (11)	8.8 (19)
Middle belt (*n*)	72.1 (80)	77.0 (47)	77.3 (34)	75.2 (85)	73.8 (76)	74.5 (161)
Highland (*n*)	20.7 (23)	13.1 (8)	11.4 (5)	17.7 (20)	15.5 (16)	16.7 (36)
MBN use (*n*)	58.6 (65)	50.8 (31)	43.2 (19)	52.2 (59)	54.4 (56)	53.2 (115)
Clinical						
Mean temperature (SD) in °C	37.7 (1.1)	37.4 (0.9)	37.6 (1.1)	37.6 (1.0)	37.6 (1.0)	37.6 (1.0)
Fever Prevalence (*n*)	52.3 (58)	50.8 (31)	40.9 (18)	48.7 (55)	50.5 (52)	49.5 (107)
Malaria prevalence (*n*)	29.7 (33)	34.4 (21)	43.2 (19)	29.2 (33)	38.8 (40)	33.8 (73)
GMPD/*µ*L of blood	7091	2805	3932	5423	4109	4658
Splenomegaly prevalence (*n*)	16.2 (18)	27.9 (17)	29.5 (13)	21.2 (24)	23.2 (24)	22.2 (48)
Mean Hb (SD) in g/dL	10 0 (2.3)	10.3 (2.6)	10.9 (2.7)	10.5 (2.8)	10.1 (2.2)	10.3 (2.5)
Anaemia prevalence (*n*)	67.6 (75)^*∗*^	63.9 (39)^*∗*^	45.5 (20)^*∗*^	60.2 (68)	64.1 (66)	62.0 (134)
Malarial anaemia prevalence (*n*)	21.6 (24)	24.6 (15)	27.5 (12)	19.5 (22)	28.2 (29)	23.6 (51)
Malnutrition (*n*)	23.4 (26)	26.2 (16)	22.7 (10)	21.2 (24)	27.2 (28)	24.1 (52)
Wasting (*n*)	4.5 (5)	6.6 (4)	9.1 (4)	1.8 (2)^*δ*^	10.7 (11)^*δ*^	6.0 (13)
Underweight (*n*)	3.6 (4)	6.6 (4)	6.8 (3)	2.7(3)	7.8 (8)	5.1 (11)
Stunting (*n*)	19.8 (22)	19.7 (12)	13.6 (6)	18.6 (21)	18.4 (19)	18.5 (40)

^*∗*^Significantly different with age (*χ* ^2^ = 6.67, *P* = 0.036).

^*δ*^Significantly different with sex (*χ* ^2^ = 5.21, *P* = 0.022).

Fever = axillary temperature ≥ 37.5°C.

Anaemia = Hb < 11 g/dL.

Malarial anaemia = MP positive + Hb < 11 g/dL.

Malnutrition = <−2 *z* score in one of the anthropometric indices (HA, WA, and WH).

**Table 2:** Prevalence of M*d*SA and M*d*SMA as affected by sociodemographic and clinical factors at presentation.

Characteristic	Category	*N*	Prevalence of M*d*SA (*n*)	*χ* ^2^	Prevalence of M*d*SMA (*n*)	*χ* ^2^
				*P* value		*P* value
Age group in years	1–5	111	41.4 (46)	3.95 0.14	14.4 (16)	0.14 0.93
	6–10	61	41.0 (25)		16.4 (10)	
	11–14	44	25.0 (11)		15.9 (7)	

Gender	Male	113	36.3 (41)	0.28 0.59	11.5 (13)	2.61 0.11
	Female	103	39.8 (41)		19.4 (20)	

SES	Poor	70	42.9 (30)	4.16 0.13	14.3 (10)	0.35 0.84
	Average	108	39.8 (43)		16.7 (18)	
	Rich	38	23.7 (9)		13.2 (5)	

Family size	1–5	107	40.2 (43)	1.32 0.52	15.0 (16)	1.66 0.44
	6–10	94	34.0 (32)		13.8 (13)	
	>10	15	46.7 (7)		26.7 (4)	

Level of education of household head	Not formal	5	60.0 (3)	8.00 0.046	0	1.98 0.58
	Primary	37	35.1 (13)		16.2 (6)	
	Secondary	94	46.8 (44)		18.1 (17)	
	Tertiary	80	27.5 (22)		12.5 (10)	

Altitude of residence	Lowland	19	63.2 (12)	8.93 0.012	26.3 (5)	3.14 0.21
	Middle belt	161	38.5 (62)		15.5 (25)	
	Highland	36	22.2 (8)		8.3 (3)	

Fever status	Febrile	107	45.8 (49)	5.25 0.019	21.5 (23)	6.33 0.012
	Afebrile	109	30.3 (33)		9.2 (10)	

Splenomegaly	Enlarged	48	39.6 (19)	0.07 0.79	14.6 (7)	0.023 0.88
	Normal	168	37.5 (63)		15.5 (26)	

Nutritional status	Malnourished	52	44.2 (23)	1.14 0.29	23.1 (12)	3.22 0.07
	Normal	164	36.0 (59)		12.8 (21)	

Total		216	38.0 (82)		15.3 (33)	

**Table 3:** Multinomial logistic regression analysis examining sociodemographic and clinical factors influencing M*d*SA and M*d*SMA.

Factors	Category	M*d*SA		M*d*SMA	
		OR (95% CI)	*P* value	OR (95% CI)	*P* value
Age group in years	1–5	0.45 (0.19–1.06)	0.07	1.08 (0.39–2.99)	0.889
	6–10	0.48 (0.19–0.122)	0.12	0.94 (0.31–2.86)	0.913
	11–14	Reference		Reference	

Gender	Male	1.07 (0.59–1.95)	0.82	1.77 (0.80–3.88)	0.16
	Female	Reference		Reference	

SES	Poor	1.32 (0.48–3.63)	0.59		
	Average	0.88 (0.43–1.78)	0.72		
	Rich	Reference		Reference	

Level of education	Not formal	3.16 (0.45–22.19)	0.22225		
	Primary	1.29 (0.19–8.74)	0.80		
	Secondary	2.26 (0.31–16.60)	0.42		
	Tertiary	Reference			

Altitude	Lowland	0.13 (0.04–0.49)	0.002	0.24 (0.05–1.21)	0.08
	Middle belt	0.38 (0.18–1.09)	0.08	0.51 (0.14–1.86)	0.31
	Highland	Reference			

Fever	Febrile	2.11 (1.45–3.87)	0.02	2.73 (1.20–6.21)	0.02
	Afebrile	Reference			

Nutritional status	Malnourished	0.76 (0.39–1.51)	0.44	0.57 (0.25–1.30)	0.18
	Normal	Reference			

**Table 4:** Variation in mean haematological values in children with M*d*SA and M*d*SMA.

Variable	Category	Number examined	Mean (SD)	*t*-test	Mean difference (95% confidence interval)
				*P* value	
WBC × 10^9^/*µ*L	M*d*SA	49	11.1 (7.2)	2.12 0.037	2.9 (0.2–5.7)
	M*d*SMA	33	8.1 (4.1)		

Lymphocyte%	M*d*SA	49	37.0 (14.3)	2.94 0.004	8.9 (2.9–14.9)
	M*d*SMA	33	28.1 (11.9)		

Granulocyte%	M*d*SA	49	50.9 (16.0)	−2.17 0.033	−7.7 (−14.7–−0.6)
	M*d*SMA	33	58.6 (15.3)		

RBC × 10^12^/*µ*L	M*d*SA	49	3.9 (1.7)	0.78 0.44	0.3 (−0.4–0.8)
	M*d*SMA	33	3.6 (0.8)		

Hb (g/dL)	M*d*SA	49	8.1 (2.0)	0.37 0.71	0.2 (−0.7–1.0)
	M*d*SMA	33	7.9 (1.8)		

Hct (%)	M*d*SA	49	26.9 (7.1)	0.43 0.67	0.6 (−2.3–3.6)
	M*d*SMA	33	26.2 (5.8)		

MCV (fl)	M*d*SA	49	74.3 (10.1)	−0.11 0.91	−0.2 (−4.5–4.0)
	M*d*SMA	33	74.5 (8.2)		

MCH (pg)	M*d*SA	49	21.7 (5.1)	−1.25 0.22	−1.2 (−3.2–0.7)
	M*d*SMA	33	22.9 (3.1)		

MCHC (g/dL)	M*d*SA	49	31.1 (2.1)	0.58 0.56	0.32 (−0.8–1.4)
	M*d*SMA	33	30.8 (2.8)		

Platelet × 10^9^/L	M*d*SA	49	233.5 (133.8)	3.37 0.001	89.9 (36.7–142.9)
	M*d*SMA	33	143.7 (86.2)		

MPV (fl)	M*d*SA	49	9.7 (1.5)	−0.97 0.34	−0.3 (−1.0–0.3)
	M*d*SMA	33	9.9 (1.4)		

PDW	M*d*SA	49	13.0 (2.9)	1.55 0.13	1.1 (−0.3–2.5)
	M*d*SMA	33	11.9 (3.0)		

RDW-CV (%)	M*d*SA	49	15.8 (3.0)	−0.80 0.43	−1.99 (−6.9–2.9)
	M*d*SMA	33	17.8 (17.0)		

RDW-SD (fl)	M*d*SA	49	43.9 (7.2)	0.77 0.44	1.1 (−1.7–3.8)
	M*d*SMA	33	42.9 (4.1)		
